# Cauliflower mosaic virus protein P6‐TAV plays a major role in alteration of aphid vector feeding behaviour but not performance on infected *Arabidopsis*

**DOI:** 10.1111/mpp.13069

**Published:** 2021-05-15

**Authors:** Quentin Chesnais, Maxime Verdier, Myriam Burckbuchler, Véronique Brault, Mikhail Pooggin, Martin Drucker

**Affiliations:** ^1^ Virus Vection, SVQV, UMR 1131 INRAE Université de Strasbourg INRAE Centre Grand Est‐Colmar Colmar France; ^2^ DEFENSIRNA, PHIM, INRAE CIRAD SupAgro IRD MUSE INRAE Centre Occitanie‐Montpellier Montferrier‐sur‐Lez France; ^3^ Present address: Insect Models of Innate Immunity, IBMC Université de Strasbourg Institut de Biologie Moléculaire et Cellulaire 2 allée Konrad Roentgen 67084 Strasbourg cedex France

**Keywords:** aphid vector, plant virus, vector behaviour, vector modification, vector transmission, viral factors

## Abstract

Emerging evidence suggests that viral infection modifies host plant traits that in turn alter behaviour and performance of vectors colonizing the plants in a way conducive for transmission of both nonpersistent and persistent viruses. Similar evidence for semipersistent viruses like cauliflower mosaic virus (CaMV) is scarce. Here we compared the effects of *Arabidopsis* infection with mild (CM) and severe (JI) CaMV isolates on the feeding behaviour (recorded by the electrical penetration graph technique) and fecundity of the aphid vector *Myzus persicae*. Compared to mock‐inoculated plants, feeding behaviour was altered similarly on CM‐ and JI‐infected plants, but only aphids on JI‐infected plants had reduced fecundity. To evaluate the role of the multifunctional CaMV protein P6‐TAV, aphid feeding behaviour and fecundity were tested on transgenic *Arabidopsis* plants expressing wild‐type (wt) and mutant versions of P6‐TAV. In contrast to viral infection, aphid fecundity was unchanged on all transgenic lines, suggesting that other viral factors compromise fecundity. Aphid feeding behaviour was modified on wt P6‐CM‐, but not on wt P6‐JI‐expressing plants. Analysis of plants expressing P6 mutants identified N‐terminal P6 domains contributing to modification of feeding behaviour. Taken together, we show that CaMV infection can modify both aphid fecundity and feeding behaviour and that P6 is only involved in the latter.

## Graphical abstract

The viral protein P6, a major determinant of symptoms in infected plants, affects aphid behaviour in a way that could be favourable for transmission of cauliflower mosaic virus.

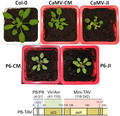

## 1 INTRODUCTION

Most plant viruses rely on arthropod vectors for their transmission to a new host. Among these, insects with a piercing‐sucking feeding behaviour such as aphids are the most efficient vectors, because they can, with great precision and without inflicting major damage to plant cells, acquire and inoculate viruses in distinct plant tissues. Evidence accumulates that viruses interact with both hosts and vectors and alter some of their characteristics to optimize transmission. The interactions may be direct, that is, changes in vector behaviour or fitness following virus acquisition by and retention in the vector, or indirect, that is, plant traits like odour, colour, and nutritive value modified by viral infection impact vector behaviour and fitness (for review see Dáder et al., 2017; Fereres & Moreno, 2009; Mauck et al., 2018). The ways viruses modify or even manipulate plant hosts and vectors depend on the transmission mode and tissue tropism of the virus (Mauck et al., 2012). Transmission modes are classified by two criteria: retention time in the vector (persistence) and vector interaction, with circulative viruses cycling through the vector body before being inoculated as a saliva component into a new host plant and noncirculative viruses interacting only with the vector mouthparts. Circulative and persistent viruses are most often phloem‐restricted and characterized by long acquisition and retention times in the vector, resulting in prolonged, often lifelong transmissibility. General predictions assume that these viruses would benefit from fast and prolonged access of vectors to the phloem, which would facilitate virus acquisition. The circulative and persistent viruses also tend to increase food quality of the host, resulting in an improvement in vector fitness and an increase in vector population (Dáder et al., 2017; Fereres & Moreno, 2009; Mauck et al., 2018). In contrast, noncirculative and nonpersistent viruses are often tissue generalists, with fast acquisition and short retention times. Plants infected with noncirculative and nonpersistent viruses may attract vectors for virus acquisition (Fereres & Moreno, 2009) and subsequently encourage them to leave the plants rapidly for fast dispersal of the virus, as shown for cucumber mosaic virus (Mauck et al., 2010). Vector departure is often related to the poor taste and low nutritive value of infected plants (Mauck et al., 2014). While many reports support these models, there are also a number of examples that do not follow the expectations. Outcome of virus–host–vector interactions may vary depending on the specific virus–host–vector combination and probably other factors (Mauck & Chesnais, 2020; Mauck et al., 2018).

Cauliflower mosaic virus (CaMV) (genus *Caulimovirus*, family *Caulimoviridae*) has features of nonpersistent and persistent viruses and is often classified as a semipersistent virus. Being a tissue generalist, CaMV can infect all cell types and is acquired from epidermis and mesophyll cells like nonpersistent viruses, but also from the phloem sap like persistent viruses (Palacios et al., 2002). With regard to vector interaction, CaMV is a noncirculative virus that binds to stylin receptors in the stylet tips of its aphid vectors (Uzest et al., 2010; Webster et al., 2018). Virus particles require the virus‐encoded transmission helper protein P2 for vector interaction that forms a complex with the virions (Leh et al., 1999; Moreno et al., 2005). Formation of this P2–virus complex, mandatory for transmission, can occur in infected plant cells or in the stylets, allowing simultaneous or sequential binding of P2 and virus to the vector (Drucker et al., 2002). Simultaneous binding is associated with fast CaMV acquisition from epidermis and mesophyll, whereas sequential binding favours uptake of CaMV from the phloem sieve tubes that contain virions but are devoid of P2 (Palacios et al., 2002).

Whereas the molecular and cellular mechanisms of CaMV acquisition are well studied, information on the effect of CaMV infection on transmission‐relevant changes in aphid behaviour and performance is scarce. Chesnais et al. (2019) reported that the green peach aphid *Myzus persicae* and the cabbage aphid *Brevicoryne brassicae* did not show any preference for CaMV‐infected *Camelina sativa* plants. Both aphid species created more intracellular punctures, ingested less phloem sap, and displayed reduced fecundity on infected plants compared to healthy plants, as expected for a nonpersistent virus.

In this study, we tested how different isolates of CaMV affect *M*. *persicae* feeding behaviour and fitness on the model plant *Arabidopsis thaliana*. We then addressed the specific role of the multifunctional viral protein P6‐TAV in modification of aphid traits. P6 is a key player in CaMV infection (for review see Pooggin & Ryabova, 2018; Schoelz et al., 2016). It is required for translation of the polycistronic CaMV 35S RNA and interacts, among others, with TOR kinase. TOR is involved in translation initiation, but plays also a pivotal role in controlling cell homeostasis of catabolic and anabolic processes. P6 binding triggers TOR phosphorylation, which then promotes protein translation and simultaneously suppresses potential antiviral autophagy and innate immunity (Hafrén et al., 2017; Zvereva et al., 2016). P6 is also a suppressor of RNA silencing and sufficient to induce typical symptoms of infection by itself (Yu et al., 2003). Finally, P6 is the matrix protein of the viral factories, where replication occurs and virus particles are stored (Schoelz & Leisner, 2017). The P6 functions in dampening plant defences and in inducing symptom expression could impact aphid–plant interactions. P6 is, therefore, a perfect candidate to modify aphid behaviour and performance.

## 2 RESULTS

### 2.1 **Effect of viral infection on *M***. ***persicae***

**FIGURE 1:**
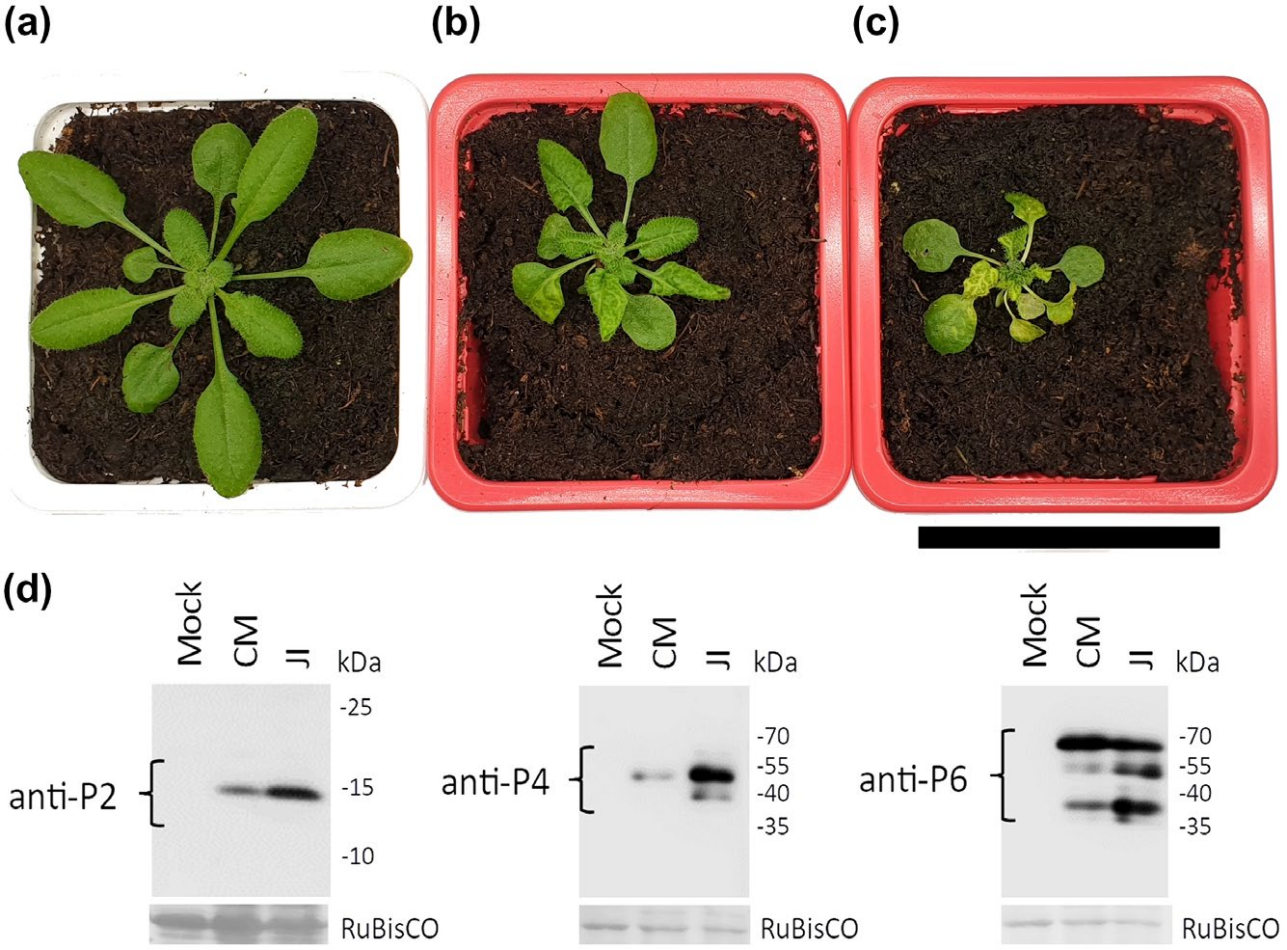
Symptoms of CaMV infection and viral protein accumulation in *Arabidopsis* Col‐0 plants. (a) Mock‐inoculated, (b) CM‐infected, and (c) JI‐infected plant 21 days postinoculation with virus‐free or viruliferous aphids. Scale bar = 5 cm. (d) Western blot analysis of accumulation of CaMV proteins in CM‐ and JI‐infected *Arabidopsis* 21 days postinoculation. The membranes were stained for P2 (left panel), P4 (middle panel), and P6 (right panel). Ponceau red staining of the large RuBisCO subunit is shown as a loading control. Molecular weights are indicated on the right of the blots

We first compared the impact of CaMV infection on *M*. *persicae* feeding behaviour and performance. For this, we chose the well‐characterized CaMV isolates Cabb B‐JI (referred to as JI) and Cm1841 that accumulate in infected Brassicaceae at high and low levels (Lung & Pirone, 1973) and cause severe and mild symptoms in *Arabidopsis*, respectively (Cecchini et al., 1998). Unlike JI, Cm1841 is a nontransmissible CaMV isolate, due to a mutation of amino acid 94 in the transmission helper protein P2 (Woolston et al., 1987). For better comparison, we reverted this mutation to wild type (wt) and carried out all experiments with the revertant virus, Cm1841‐Rev, hereafter referred to as CM. Figure 1a–c shows that, at 21 days postinoculation, JI‐infected *Arabidopsis* Col‐0 plants displayed stronger symptoms compared to CM‐infected plants with a reduced growth, intense yellowing, and leaf curling. In addition, we observed lower accumulation of P2, P4 capsid protein, and P6‐TAV protein in CM‐infected plants (Figure 1d).

**FIGURE 2:**
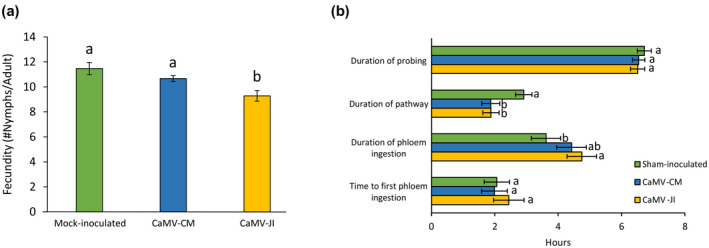
Fecundity and feeding behaviour of *Myzus persicae* on mock‐inoculated or CaMV‐infected *Arabidopsis*. Two‐week‐old plants were inoculated with the indicated isolate and used 3 weeks later for the experiments. (a) Aphid fecundity after 5 days of infestation (*n* = 28–33). (b) Aphid feeding behaviour parameters recorded with the electrical penetration graph (EPG) technique (*n* = 22–27). Letters show significant differences between plant infection status as tested by GLM followed by pairwise comparisons using “emmeans” (*p* < .05; method: Tukey)

We next evaluated aphid fecundity on infected and mock‐inoculated plants by counting the number of offspring of synchronized adult wingless aphids after 5 days of infestation. Fecundity was significantly lower on JI‐infected *Arabidopsis* compared to mock‐inoculated control plants and CM‐infected plants (generalized linear model [GLM], *df* = 2, χ^2^ = 15.542, *p* < .001; Figure 2a).

Then we assayed aphid feeding behaviour by the electrical penetration graph (EPG) technique. Behaviour of individual aphids was recorded for 8 hr on mock‐inoculated or infected *Arabidopsis*. The total duration of probing in plant tissue and the time to the first phloem phase were not affected by either CaMV isolate (GLM, *df* = 2, χ^2^ = 0.952, *p* = .621; Cox model, χ^2^ = 0.07, *p* = .96; Figure 2b). The duration of the pathway phase was significantly reduced on infected *Arabidopsis* for both isolates (GLM, *df* = 2, χ^2^ = 11.14, *p* = .004). The duration of phloem ingestion was significantly longer on JI‐infected *Arabidopsis* compared to CM‐infected and mock‐inoculated plants (GLM, *df* = 2, χ^2^ = 6.27, *p* = .04; Figure 2b).

### 2.2 Effect of P6 on aphids

**FIGURE 3:**
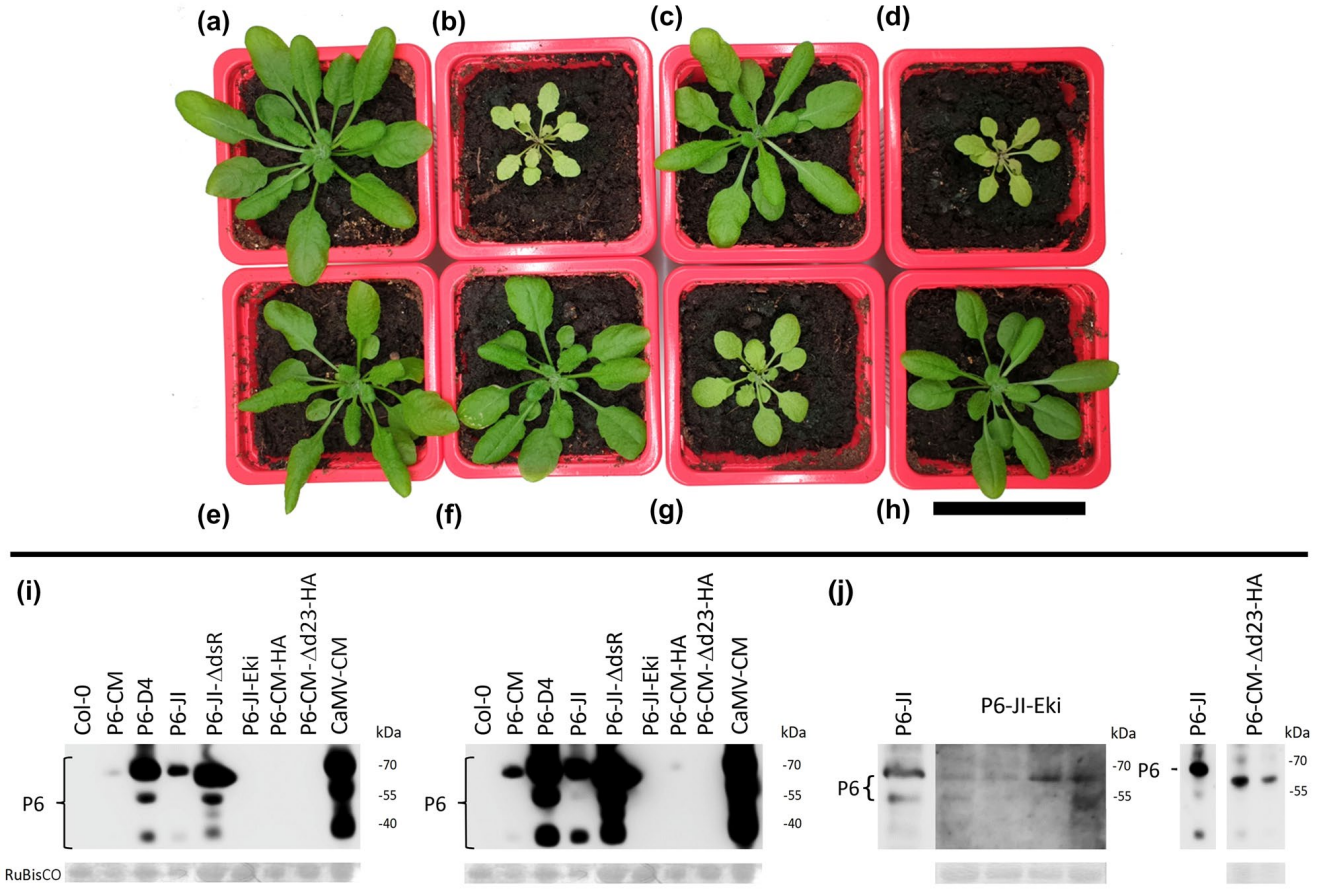
Phenotype of 5‐week‐old transgenic *Arabidopsis* plants expressing various P6 proteins. The images show (a) an untransformed Col‐0 plant and (b–h) transgenic Col‐0 plants expressing (b) P6 from the CaMV isolate CM; (c) P6 from isolate D4; (d) P6 from isolate JI; (e) the P6 dsR domain deletion mutant from isolate JI, referred to as JI‐ΔdsR; (f) the P6 Eki mutant from isolate JI, referred to as JI‐Eki; (g) HA‐tagged wild‐type P6 from CM (CM‐HA), and (h) the HA‐tagged P6 Vir/Avr domain deletion mutant from isolate CM, referred to as CM‐Δd23‐HA. Scale bar = 5 cm. (i) Western blot analysis of P6 protein accumulation in 5‐week‐old transgenic plants expressing the indicated P6 mutants. The figure shows the same blot revealed with a short (left) or a longer (right) exposure time to visualize weak bands. Extracts prepared from untransformed Col‐0 and from CM‐infected leaves were loaded as negative and positive controls, respectively. (j) Detection of P6‐JI‐Eki and P6‐CM‐Δd23‐HA by western blot. The two proteins that were not detected in the blot shown in (i) could be revealed in a different experiment using more concentrated extracts. Each lane presents extract from a different transgenic plant. Signals from wild‐type P6 loaded on the same blots are shown to the left of the panels and either the blots were exposed much shorter (P6‐JI‐Eki) or extracts were diluted (P6‐CM‐Δd23‐HA). Note that the mutant P6 concentration varied considerably. Ponceau red staining of the large RuBisCO subunit is shown as a loading control

We used transgenic *Arabidopsis* expressing wt and mutant P6 proteins from the constitutive 35S promoter to determine whether P6 is involved in plant–aphid interactions. Figure 3a–h shows the phenotype of Col‐0 plants expressing the P6 proteins 5 weeks after germination: Plants expressing wt P6 from JI or CM or P6 from CM with an N‐terminal HA‐tag (P6‐CM‐HA) were smaller and leaves showed yellowing but no leaf curling as CaMV‐infected plants (Figure 3b,d,g). Plants expressing P6 from CaMV isolate D4 resembled untransformed plants (Figure 3c). *Arabidopsis* plants expressing mutated versions of P6 from isolate JI, JI‐Eki (bearing three amino acid substitutions at P6’s N‐terminus) and JI‐ΔdsR (containing a deletion of the TOR‐ and RNA‐binding region), displayed no or only a weak phenotype compared to untransformed plants (Figure 3e,f). Also, the P6 CM‐Δd23‐HA mutant (containing an N‐terminal HA‐tag and a deletion of the virulence/avirulence region Vir/Avr) induced no visible phenotype (Figure 3h). Western blotting indicated strong accumulation of P6‐D4 and P6‐JI‐ΔdsR and low accumulation of P6‐CM and P6‐JI in transgenic plants (Figure 3i). P6‐CM‐HA was visible after prolonged exposure of the blot, and P6‐JI‐Eki and P6‐CM‐Δd23‐HA only after redoing the blots (Figure 3j).

**FIGURE 4:**
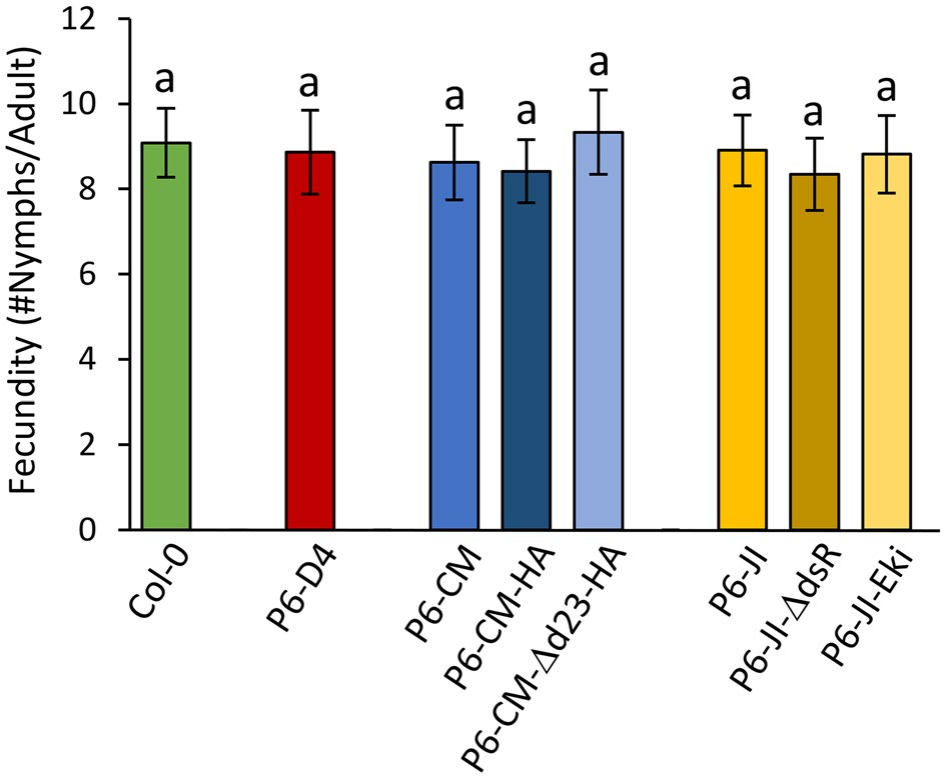
*Myzus persicae* fecundity 5 days after deposit on untransformed or transgenic 5‐week‐old *Arabidopsis* plants expressing P6 proteins. Different letters show significant differences between plants as tested by GLM followed by pairwise comparisons using “emmeans” (*p* < .05; method: Tukey, *n* = 21–24). No statistically significant differences were recorded

*M. persicae* fecundity was assayed on the P6‐expressing transgenic plants. In contrast to CaMV‐infected plants, aphid fecundity was not affected on any transgenic plant when compared to untransformed plants (GLM, *df* = 7, χ^2^ = 6.61, *p* = .478; Figure 4).

**FIGURE 5:**
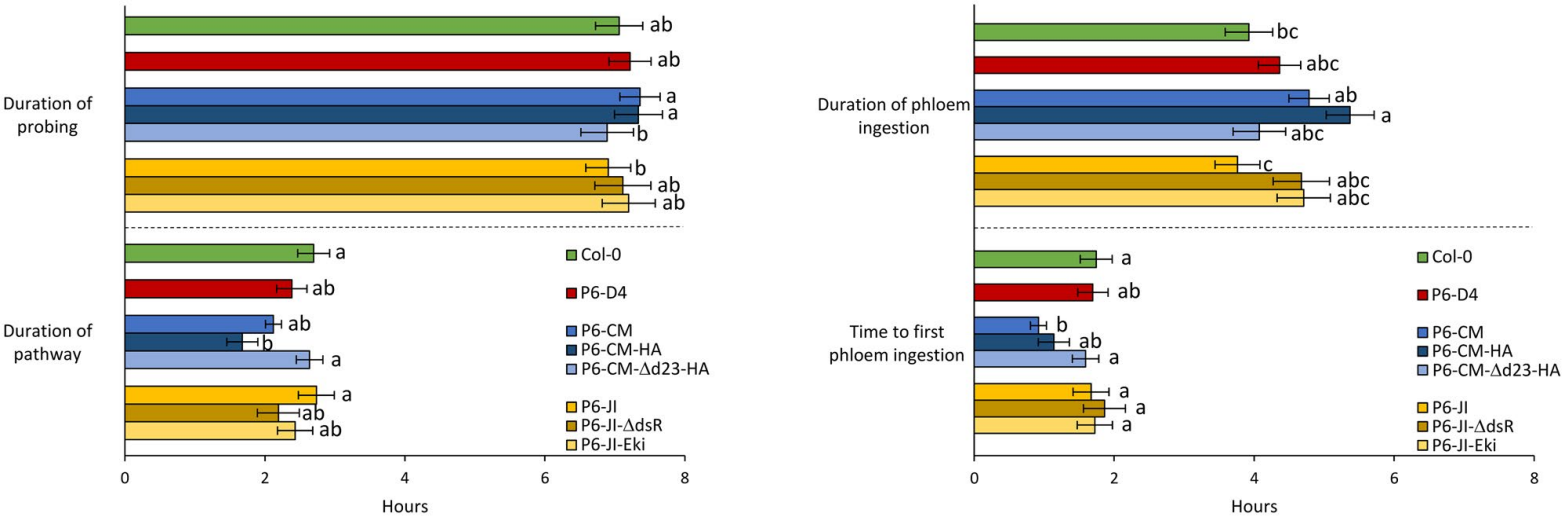
Aphid feeding behaviour parameters recorded by EPG on untransformed Col‐0 and transgenic P6‐expressing 5‐week‐old *Arabidopsis*. Different letters indicate significant differences between plants as tested by GLM followed by pairwise comparisons using “emmeans” (*p* < .05; method: Tukey, *n* = 25–31)

Then, the feeding behaviour of *M*. *persicae* on the P6‐transgenic plants was analysed by EPG (Figure 5). All aphid behaviour parameters on P6‐D4 plants were similar to those on untransformed Col‐0 plants. We detected significant differences in aphid feeding behaviour on the other transgenic plants. Compared to plants expressing P6‐CM and P6‐CM‐HA, aphids probed for significantly shorter times on P6‐CM‐Δd23‐HA and P6‐JI plants (GLM, *df* = 7, χ^2^ = 23.07, *p* = .002; Figure 5). The pathway phase was significantly reduced on P6‐CM‐HA plants, compared to P6‐CM‐Δd23‐HA, JI, and untransformed Col‐0 plants (GLM, *df* = 7, χ^2^ = 24.33, *p* < .001). The duration of phloem ingestion was significantly longer on P6‐CM‐HA plants compared to P6‐JI and untransformed plants (GLM, *df* = 7, χ^2^ = 25.12, *p* < .001). Aphids reached the first phloem phase significantly faster on plants expressing P6‐CM compared to untransformed Col‐0 or plants expressing P6‐JI and P6‐CM‐Δd23‐HA (GLM, *df* = 7, χ^2^ = 20.76, *p* = .04).

## 3 DISCUSSION

Many studies report changes in vector behaviour and performance after infection of plants with viruses, but only a few identify viral determinants involved in vector manipulation. Also, most work centres on persistent and nonpersistent viruses; studies on semipersistent viruses are scarce. To fill this gap, we studied the feeding behaviour and fecundity of aphids on *Arabidopsis* infected with a severe and a mild isolate of CaMV, a virus with transmission properties of both nonpersistent and persistent viruses. Then, we studied the potential role of the multifunctional viral protein P6 in virus‐induced alterations of host‐plant traits and vector responses.

### 3.1 Effect of CaMV infection on aphid fecundity

The severe and high‐accumulating CaMV isolate JI reduced aphid fecundity on infected *Arabidopsis* significantly (c.2.5 fewer nymphs produced per adult after 5 days compared to mock‐inoculated plants), while infection with the mild and low‐accumulating isolate CM had only a moderate impact on aphid fecundity, below the significance threshold (c.1 nymph less produced per adult after 5 days compared to mock‐inoculated plants; Figure 2a). Thus, both increased symptom severity and higher viral accumulation correlated with reduced aphid fecundity. Whether symptom severity is related to CaMV accumulation is still unclear and seems to depend on the host–virus association (Doumayrou et al., 2013). Lower fecundity and other negative effects of plant infection on vector performance such as reduced vector survival or delayed development are predicted for nonpersistent viruses. These modifications may promote rapid dispersion of vectors and subsequently virus transmission (Mauck et al., 2018). Indeed, mathematical models show that increasing departure rates from hosts lead to increased vector movements between host plants, which is beneficial for virus dissemination (Carr et al., 2020; Shaw et al., 2017). Whether this applies for CaMV on *Arabidopsis* remains to be determined because although reduced fecundity is a proxy for aphid fitness, it does not indicate that aphids will really leave CaMV‐infected plants faster than healthy plants, as for example documented for *M*. *persicae* on cucumber mosaic virus‐infected squash (Mauck et al., 2010). A negative effect of CaMV JI on *M*. *persicae* fecundity was already observed by Chesnais et al. (2019), albeit on another host plant, the Brassicaceae member *Camelina sativa*. This suggests that the effect on fecundity is not host plant‐specific, but rather virus isolate‐specific.

### 3.2 Effect of CaMV infection on aphid feeding behaviour

*M. persicae* on CaMV‐JI‐infected plants spent significantly less time in the pathway phase in the leaf tissue and more time ingesting phloem sap than aphids on healthy plants (Figure 2b). This is expected to be counterproductive for transmission of nonpersistent viruses (Carr et al., 2020; Eigenbrode et al., 2018; Mauck et al., 2012). In fact, typical nonpersistent viruses like potyviruses or cucumber mosaic virus (single‐stranded RNA viruses of families Potyviridae and Bromoviridae, respectively) are acquired during short intracellular stylet punctures in plant epidermis and mesophyll (Martin et al., 1997). They are lost from the stylets when aphids stay on plants for longer times and when the stylets reach the sieve tubes that do not contain transmission‐competent virus forms (Kloth & Kormelink, 2020; Wang & Ghabrial, 2002). The case is different for CaMV that is acquired efficiently from both cells and phloem sap and after both short and long acquisition periods (Bouchery et al., 1990; Markham et al., 1987; Palacios et al., 2002). Therefore, both improved palatability (characterized by few probes, rapid access to the phloem, and long phloem ingestion) and reduced palatability (characterized by many probes, impairment in phloem access, and reduced phloem ingestion) of infected plants can be conducive for CaMV transmission. In fact, Chesnais et al. (2019) observed, in contrast to our results here, a significantly lower number of probes and an increased sap ingestion of *M*. *persicae* feeding on CaMV‐JI‐infected *Camelina* plants. Yet transmission efficiency is high using either plant species as a virus source (Dáder et al., 2019; Verdier, 2020).

In summary, CaMV is, with regard to transmission, a special case combining characteristics of nonpersistent and persistent transmission modes. We propose that the particular acquisition mode of CaMV optimizes transmission by lowering its dependency on specific aphid feeding behaviours. Most noncirculative viruses (i.e., nonpersistent and semipersistent viruses) are transmitted by a large number of vectors (e.g., 27 for CaMV, 89 for TuMV; Edwardson & Christie, 2018; Kennedy et al., 1962) while circulative viruses are generally transmitted by less than a dozen. This means that these noncirculative viruses need to interact with multiple vectors, and it is unlikely that they can engage in manipulations that are specific for each vector species. Rather, they must target a common and accessible vector feature, for example a conserved receptor in the aphid stylets (Webster et al., 2018), to which they can bind easily and already after short vector–plant contact. This enables most aphids, even noncolonizers, to engage in behaviours conducive with nonpersistent virus acquisition and inoculation (e.g., epidermis and mesophyll probing). As a consequence, there may be little (or no) advantage and no strong selection pressure for CaMV and other noncirculative viruses to manipulate vector feeding behaviours (Mauck & Chesnais, 2020).

### 3.3 A role for P6 in aphid manipulation

Aphid fecundity was unchanged on *Arabidopsis* plants expressing wt P6 from JI or CM (Figure 4), although these plants displayed dwarfing and bleaching, indicative of physiological modifications (Figure 3b,d). Three nonexclusive explanations are possible. The first one is that P6 alone is not involved in modifying aphid fecundity. This would mean that other viral determinants (proteins or RNAs), either independently or in concert with P6, induce changes in plants that reduce aphid fecundity. Indeed, it has been suggested that the CaMV proteins P1–5 are mainly responsible for leaf malformation, while P6 causes chlorosis and dwarfism (Yu et al., 2003). The second explanation is that P6 levels in the transgenic plants were too low to reduce aphid fecundity. Indeed, we observed that P6 levels were considerably lower and variable in transgenic plants compared to infected plants (Figure 3i,j). However, in CaMV‐infected plants a major fraction of P6 protein is located in inclusion bodies/replication factories (Schoelz & Leisner, 2017) and only a small fraction of P6 may be available in a soluble form to fulfil its other functions (reviewed by Pooggin & Ryabova, 2018). Finally, it is also possible that phloem sap composition (e.g., amino acid and/or sugar concentration and composition) was changed in infected but not in transgenic plants. Further experiments are required to solve this issue.

Some aphid feeding behaviour parameters were significantly altered on transgenic plants expressing P6 (Figure 5). Compared to control plants, the time to first phloem ingestion was reduced on transgenic P6‐CM plants. Interestingly, no such effect was observed on plants infected with CaMV‐CM (Figure 2b). This might indicate that other viral determinants counteracted the effect of P6. A second significantly changed parameter was the duration of phloem ingestion, which was longer on P6‐CM plants than on P6‐JI plants. Opposite results were obtained with infected plants, where the duration of phloem ingestion was longer on JI‐infected than on mock‐inoculated plants, with the duration of CM‐infected *Arabidopsis* being intermediate. Again, this observation indicates that P6 contributes to the modifications in phloem sap ingestion, but that other viral determinants are involved as well.

### 3.4 P6 domains involved in aphid manipulation

**FIGURE 6:**
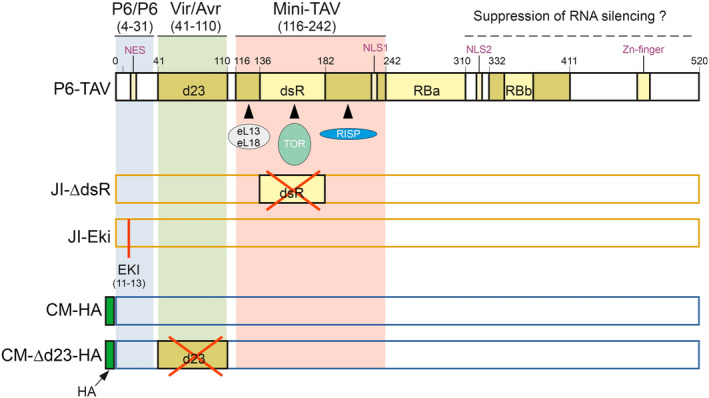
Functional domains of P6 and P6 mutants used in this study. Only relevant domains and interacting proteins are shown. The N‐terminal half of P6 contains three major regions: The N‐terminus (amino acids 4–31) contains one of several P6/P6 self‐interaction domains required for P6’s function as the matrix protein of virus inclusions. This region is followed by the Vir/Avr domain delimited by the d23 region, which is involved in chlorosis induction and dwarfism. The mini‐TAV domain comprises amongst others an N‐terminal region binding to ribosomal proteins eL13 and eL18, the central double‐stranded RNA‐binding (dsR) region that interacts with TOR kinase, and a C‐terminal region that interacts with reinitiation supporting protein (RISP) and contains also a nuclear localization signal (NLS1). The mini‐TAV region is important for antiviral autophagy, innate immunity, and polycistronic translation. One of the functions of the C‐terminal half of P6 is probably suppression of RNA silencing, in which also the N‐terminal nuclear export signal (NES) and the two nuclear localization signals (NLS1 in the mini‐TAV domain and NLS2 in the C‐terminal region) play a role. The dsR region is deleted in P6‐JI‐ΔdsR. In P6‐JI‐Eki, the conserved Eki motif just preceding the NES is substituted by three alanines. CM‐HA contains an N‐terminal HA‐tag as modification and CM‐Δd23‐HA in addition a deletion of the d23 region. RBa, RBb, RNA‐binding regions. Figure adapted from Pooggin and Ryabova (2018)

The comparison of the aphid feeding behaviour on transgenic *Arabidopsis* expressing various P6 versions yielded evidence for the involvement of its N‐terminus in modification of plant–aphid interactions (Figure 5). P6 from the D4 isolate, although accumulating to much higher levels in transgenic plants than P6‐JI or P6‐CM (Figure 3i), had no effect on aphid behaviour, and plants displayed a wt phenotype (Figure 3c), as previously reported by Yu et al. (2003). Because P6‐D4’s RNA silencing suppression domains are functional in *Arabidopsis* (Shivaprasad et al., 2008; Zvereva et al., 2016), we conclude that the silencing suppression domain (probably located in the C‐terminal portion of P6; see Figure 6 for an overview of P6’s functional domains) has no impact on aphid behaviour or on symptom expression. P6‐D4’s Vir/Avr domain, responsible for symptom expression, and the mini‐TAV domain, required for translation transactivation and TOR‐mediated immunity, are nonfunctional in *Arabidopsis* (Yu et al., 2003; Zvereva et al., 2016), precluding any definitive conclusions but leaving a possibility that corresponding domains of P6‐CM and P6‐JI can contribute to modification of aphid feeding behaviour. Indeed, aphids spent more time in the pathway phase on P6‐CM‐Δd23‐HA plants (containing an N‐terminal HA‐tag and a deletion of the Vir/Avr domain), compared to P6‐CM‐HA plants, despite similar low accumulation in transgenic plants. Compared to untransformed plants, aphids had a shorter pathway phase and longer phloem sap ingestion on P6‐CM‐HA but not on P6‐CM‐Δd23‐HA plants. This is in favour of a role of the Vir/Avr region in altering aphid feeding behaviour. Neither P6‐JI nor P6‐JI‐ΔdsR plants (functional Vir/Avr domain, no TOR interaction) had a significant effect on aphid behaviour, despite higher protein accumulation in transgenic plants compared to P6‐CM‐Δd23‐HA. This might indicate that the effect of the Vir/Avr domain on aphid behaviour is CaMV isolate‐specific. In fact, the Vir/Avr domain is one of the most variable domains of P6 among CaMV isolates. Notably, despite previous controversial evidence (Laird et al., 2013), the P6‐CM Vir/Avr domain was found to be crucial for virus infectivity and virulence but not suppression of RNA silencing in *Arabidopsis* (Zvereva et al., 2016). Moreover, this domain contributes to suppression of salicylic acid‐dependent autophagy and effector‐triggered innate immunity in *Arabidopsis*, although it was not absolutely essential for this TOR‐dependent function of P6‐CM (Zvereva et al., 2016). We speculate P6‐mediated dampening of pattern‐ and/or effector‐triggered innate immunity may explain the observed changes in aphid feeding behaviour. Indeed, aphids are known to induce innate immunity responses in *Arabidopsis* and deliver effector proteins to suppress these responses (Mugford et al., 2016; Prince et al., 2014). A role for the P6 mini‐TAV region with TOR‐binding domain in modification of aphid behaviour remains disputed. Despite its high accumulation levels in transgenic plants, comparable to those in virus‐infected plants, P6‐JI‐ΔdsR (no TOR interaction) only slightly affected aphid behaviour. This indicates that the TOR‐binding domain is not required but other elements of the mini‐TAV region preserved in the mutant might still be important for modification of aphid behaviour. Finally, P6‐JI‐Eki did not change aphid behaviour significantly, suggesting that the extreme N‐terminus of P6 may not be involved in aphid interactions. However, in this case the mutant protein accumulated in transgenic plants at extremely low levels, precluding any definitive conclusion.

Taken together, this report shows that aphid responses to CaMV infection depend on the virus isolate, and that part of the effects, but not the entire response, relies on P6.

## 4 EXPERIMENTAL PROCEDURES

### 4.1 Cloning

Cm1841‐Rev, where the mutation of amino acid 94 in P2 in the Cm1841 genome is reverted to wt, was obtained by site‐directed mutagenesis using the QuikChange Lightning Kit (Agilent) following the manufacturer’s instructions and using the oligonucleotides QuikReve‐F (5′‐GTCAGTTTTTAATACTGCAAAAAACATTTTTAAAAGTGGGGGGGTTGATTACTCG‐′) and QuikReve‐R (5′‐CGAGTAATCAACCCCCCCACTTTTAAAAATGTTTTTTGCAGTATTAAAAACTGAC‐3′). The two oligonucleotides contain a silent G → A mutation at nucleotide 277 of the P2 sequence to create a *Dra*I restriction site for easy identification of recombinants and an A → G mutation at nucleotide 280 of P2 to revert amino acid R_94_ to wt G_94_. The pCa122 plasmid containing 1.2 copies of the Cm1841 genome (Tsuge et al., 1994) was used as a matrix. The cycling programme was 2 min at 95 °C; followed by 18 cycles of 20 s at 95 °C, 10 s at 60 °C, and 7 min at 69 °C; and a final extension step of 5 min at 68 °C. The plasmid pCa122rev was verified by sequencing the mutated region.

### 4.2 Virus inoculation

Initial inoculation was performed with the pCa122rev plasmid or the pGreen‐BJI plasmid (Khelifa et al., 2010) coding for the Cabb B‐JI genome. The plasmids were rub‐inoculated with carborundum into the first true leaf of turnip (*Brassica napus* ‘Just Right’) seedlings (20 μg plasmid per seedling at the 2‐leaf stage). When disease symptoms were well developed, leaves were harvested and stored at −20 °C. The virus was passaged once by mechanical inoculation of *Arabidopsis* ecotype Col‐0 plants at the 4‐ to 8‐leaf stage. For this, frozen turnip leaves were thawed and ground in a mortar with carborundum in 10 mM HEPES buffer pH 8.0 at a ratio of 1 g leaf per 0.5 ml buffer, and used for rub inoculation. All further inoculations were by aphid transmission using infected *Arabidopsis* plants as virus source. Aphids were deposited on CaMV‐infected plants overnight, and then five or six viruliferous aphids were placed on 2‐week‐old *Arabidopsis* for a few hours before being removed manually.

Plants infected with Cm1841‐Rev (CM) or with Cabb B‐JI (JI) were used 3 weeks after inoculation for experiments. Growth conditions were 8 hr light/16 hr dark at 22/20 °C.

### 4.3 *Arabidopsis* mutant lines

The transgenic lines P6‐CM‐HA and P6‐CM‐Δd23‐HA are described by Zvereva et al. (2016), P6‐CM1841 (as lines CM‐6 and CM‐8) and P6‐D4 (as D4‐2) by Yu et al. (2003), and P6‐JI (as AT7) and P6‐JI‐ΔdsR (as AT7ΔdsR) by Schepetilnikov et al. (2011). The P6‐JI‐Eki line is described as TAVm3 by Haas et al. (2008). Wt and mutant lines were all in the Col‐0 background.

### 4.4 Aphid rearing

The *M. persicae* green peach aphid clone was originally isolated in the Netherlands. Aphids were reared on Chinese cabbage (*Brassica rapa pekinensis*) in a growth chamber at 20 ± 1 °C, under a 16 hr photoperiod. Only wingless forms were used in assays. For synchronization, we placed adults on detached Chinese cabbage leaves that were spread on 1% agarose in a Petri dish. The adults were removed 24 hr later and the newborn larvae used in experiments after another 7 days when they had reached the adult stage.

### 4.5 Aphid feeding behaviour

We used the electrical penetration graph DC‐system as described by Tjallingii (1988) to investigate the effects of plant CaMV infection on the feeding behaviour of *M*. *persicae*. Eight aphids were connected to the Giga‐8 DC‐EPG amplifier and each one was placed on the leaf of an individual *A. thaliana* plant. The recordings were performed continuously for 8 hr during the photophase. Each aphid–plant system was placed inside a Faraday cage at 21 ± 1 °C. Acquisition and analysis of the EPG waveforms were carried out with PROBE 3.5 software (EPG Systems). Relevant aphid behaviour EPG parameters were calculated with EPG‐Calc 6.1 software (Giordanengo, 2014) and were based on different EPG waveforms described by Tjallingii and Hogen Esch (1993). The following parameters were selected because they are relevant and important for the acquisition of CaMV by aphids: the total duration of “probing time”, “pathway phase”, and “phloem sap ingestion phase” and the time needed by the aphid to reach the phloem. For each condition (healthy or infected and wt or mutant, respectively), EPGs of 25–30 individual aphids were analysed. Aphids that produced signals (i.e., total duration of probing time) for fewer than 5 hr were excluded from the analysis.

### 4.6 Aphid fecundity

Synchronized wingless adults (8 ± 1 days old) were randomly selected from the aphid pools and transferred onto *Arabidopsis* plants (one aphid per plant) to study adult fecundity. The number of nymphs produced were recorded after 5 days. Adult aphids that died before day 5 were excluded from the analysis. Data on CaMV‐infected or mock‐inoculated *Arabidopsis* were collected in three repetitions, and data on *Arabidopsis* mutants were collected in four repetitions, comprising altogether 21–33 aphids per condition (infected or mock‐inoculated and wt or mutant, respectively).

### 4.7 Statistical analysis

We used GLMs with a likelihood ratio and the chi‐square (χ^2^) test to assess whether there was an effect of plant infection or *Arabidopsis* mutants on *M*. *persicae* feeding behaviour. When a significant effect was detected, a pairwise comparison using estimated marginal means (R package “emmeans”; *p* value adjustment with Tukey method) at the .05 significance level was used to test for differences between treatments. Data on aphid feeding behaviour (probing, pathway, and phloem sap ingestion phases) were not normally distributed; accordingly we carried out a GLM using a gamma (link = “inverse”) distribution. Data on aphid feeding behaviour (t1 < E2) were modelled using the Cox proportional hazards model and we treated cases where the given event did not occur as censored. The assumption of validity of proportional hazards was checked using the functions “coxph” and “cox.zph”, respectively (R package “survival”). When a significant effect of one of the main factors was detected or when an interaction between factors was significant, a pairwise comparison using estimated marginal means (R package “emmeans”) (*p* value adjustment with Tukey method) at the .05 significance level was used to test for differences between treatments. The fit of all GLMs was controlled by inspecting residuals and QQ plots. All statistical analyses were performed using R software v. 3.3.2 (www.r‐project.org/).

### 4.8 Western blot

Total leaf extracts were prepared as follows: Leaves were frozen in a mortar with liquid N_2_ and ground with a pistil to a fine powder. The powder was transferred to a 1.5 ml reaction tube and 2× Laemmli buffer (Laemmli, 1970) was added at a ratio of 1:1 (wt/vol). The samples were heated for 5 min at 80 °C and centrifuged for 10 min at 16,000 × g, and aliquots of the supernatants were charged on 6/12.5% discontinuous sodium dodecyl sulphate (SDS)‐polyacrylamide gels. Proteins were transferred after SDS polacrylamide gel electrophoresis onto nitrocellulose membranes using the wet blotting technique (Towbin et al., 1979). Efficiency of transfer was controlled by Ponceau red staining. Membranes were blocked for 30 min with 5% low‐fat milk powder in Tris‐buffered saline (TBS) and incubated overnight at 4 °C with primary antibodies. After three washes with TBS, membranes were incubated for 3–4 hr at room temperature with secondary antibodies. After another three washes with TBS, protein bands were visualized by enhanced chemiluminescence using a G‐Box. The following 1:2,000 dilutions of primary antibodies were used: anti‐P2 (Blanc et al., 1993), anti‐P4 (Champagne et al., 2004), and anti‐P6 (Khelifa et al., 2010). Secondary antibodies were horseradish peroxidase conjugates, which were used at a 1:25,000 dilution.

## Data Availability

The data that support the findings of this study are available from the corresponding author upon reasonable request.
